# Meta-analysis of a mindfulness yoga exercise intervention on depression – based on intervention studies in China

**DOI:** 10.3389/fpsyg.2023.1283172

**Published:** 2023-12-11

**Authors:** Yuehang Yang, Dawei Cao, Teng Lyu, Wei Gao

**Affiliations:** ^1^College of Physical Education, Huaibei Normal University, Huaibei, China; ^2^College of Literature, Huaibei Normal University, Huaibei, China; ^3^Sports Department, Huaibei Institute of Technology Sports Department, Huaibei, China

**Keywords:** anxiety, depression, meta-analysis, mindfulness thinking, yoga exercises

## Abstract

**Background:**

Using statistical methods to analyze and summarize the research data of the inclusion criteria, to provide a quantitative average effect size to interpret the influence of mindfulness yoga exercise on patients with different depressive symptoms, explain the therapeutic effect of mindfulness yoga therapy on depression and its possible mechanism of action, and provide new ideas for the clinical treatment of patients with depression.

**Method:**

Review Manage 5.4 software was used to comprehensively evaluate the effect of yoga exercise on depression interventions to provide a reference for improving mental health. CNKI, PubMed, Web of science, EBSCO were searched for all case–control research articles on yoga for depression from 2000 to 2022. After screening, data extraction and quality evaluation of randomized controlled trials (RCTs) by inclusion and exclusion criteria, a total of 22 studies with 2,216 patients were included, including 1,101 in the yoga intervention group and 1,115 in the control group.

**Results:**

The results showed a large heterogeneity in the literature on the effect of yoga exercise on depression, with a combined total effect size [*SMD* = −1.53, 95%*CI* (−1.96, −1.10), *p* < 0.00001].

**Conclusion:**

Mindfulness yoga exercise is effective in preventing and treating depression and improving mental health, and may be considered as a non-medical, low-cost intervention as an adjunct to pharmacological treatment.

## 1 Introduction

With the acceleration of the social development process, the phenomenon of sub-health is frequent, and while the public is concerned about the physical health of the whole population, mental health has become an urgent problem. The National Depression Blue Book 2022 China Mental Health Survey shows that there are currently 95 million people suffering from depression in China, and about 280,000 people commit suicide every year, 40% of whom suffer from depression (China Education and Research Network, 2022). According to the World Health Organization (WHO), an estimated 3.8% of the population experience depression, including 5% of adults (4% among men and 6% among women), and 5.7% of adults older than 60 years. Approximately 280 million people in the world have depression. Depression is about 50% more common among women than among men. Worldwide, more than 10% of pregnant women and women who have just given birth experience depression. More than 700,000 people die due to suicide every year (World Health Organization, 2023). The global burden of mental disorders has increased after the Newcastle pneumonia epidemic, with cases of major depression and anxiety disorders increasing by 28 and 26%, respectively, and a surge of 53 million people with depression, an increase of 27.6% (Santomauro et al., 2021). A recent WHO report shows that in the first year of the new pandemic, the global incidence of anxiety and depression increased significantly by 25 percent. Nearly 1/3 of the residents who were isolated at home experienced varying degrees of depression, anxiety, insomnia and acute stress, and more than 10% of them failed to fully recover after the pandemic.

When diagnosed with anxiety- or sleep-related depression, patients take medications such as antidepressants and benzodiazepines; the risk of recurrence is about 50%. This pathology and its chemotherapy can affect individual health and life balance, such as cognitive impairment, family and work. In addition, side effects may produce dependence, inability to concentrate or drive, to inhibit suicide attempts (Kuyken et al., 2015).

Compared with previous studies, the causes of depression are multidimensional, including psychological factors (personal history, loss, trauma), biological factors (genetic susceptibility, neurochemical disorders, bacteria) and environment (stress, social interaction, family environment, physical environment). Different degrees of depression will show different characteristics. In mild depression: appearance as usual, inner pain experience, sadness, distress, depression. Moderate depression can have low mood, sad face, sigh, low self-esteem and other states, some patients are often accompanied by neurosis symptoms, such as: inattention, memory loss, slow response, insomnia and dreaminess and other symptoms. With severe depression, there will be pessimism, despair, self-blame, delusion, loss of appetite, weight loss, dysfunction, and severe suicide attempts, and even suicidal behavior (Xu, 2021). It poses a serious threat to health, so we must attach great importance to it and treat it in time. As a long-term continuous state, depression can be treated by psychological counseling combined with physical exercise, and its positive effect is more lasting and safe. Exercise can secrete endorphins and dopamine in the brain, thus effectively relieving stress. After the patient releases the pressure and relieves the mood, it is more conducive to continuing clinical treatment, improving the body’s immunity and improving the therapeutic effect.

The yoga of mindfulness is a combination of “mindfulness practice” and “hatha yoga,” which teaches patients to practice “hatha yoga” while looking at the phenomena of the body and mind in the present moment (Yao et al., 2023). In recent years, some scholars have found that the combination of mindfulness and yoga exercises has a releasing effect on non-severe depression patients. The aim of this study is to systematically evaluate and apply meta-analysis to determine the effect of yoga treatment on depressed patients, in order to draw objective and accurate conclusions and provide a theoretical reference for the future application of yoga in mental health treatment and to promote the integration of somatic medicine.

### 1.1 Mindfulness yoga features and benefits

Mindfulness-based stress reduction (MBSR) is a systematic meditation training method based on positive thinking, which commonly includes positive meditation, body scanning and positive movement training (positive walking, positive yoga) (You, 2020). Among them, mindfulness yoga is derived from the asanas of hatha yoga, which incorporates the skill of truthfulness in positive thinking into yoga postures, focusing on the integration of present moment awareness with the body, observing the reality of mind and body in the present moment, and accepting it as it is (Miao et al., 2023a). Gentle and soothing, the long-term practice of positive yoga can improve one’s physical, mental, emotional and spiritual abilities, and is a form of exercise that achieves harmony and unity between body, mind and spirit. As awareness continues to increase, tolerance increases and acceptance and tolerance strength will slowly increase (Sun and Wang, 2020). In addition, positive yoga has multiple benefits such as improved blood sugar, improved cardio-respiratory and muscle strength, and improved quality of life, and no adverse effects such as acute injuries have been observed.

Studies have shown that in Major depressive disorder, positive yoga exercise is as effective as antidepressant medication and is associated with reduced severity of depressive symptoms and increased treatment remission (Guerrera et al., 2020). Xu et al. (2023) confirmed that in patients with mild to moderate Parkinson’s disease, a positive yoga program was associated with improving motor dysfunction and mobility, reducing anxiety and depressive symptoms, and improving mental health and quality of life, the intervention was superior to stretching and resistance training programs. James-Palmer reviewed 27 studies involving youth with different health conditions and found that overall 70% of studies showed significant improvement; 58% of studies assessing anxiety and depression of the studies showed a reduction in both symptoms, and 25% showed a reduction in anxiety only. In addition, 70% of the studies assessing anxiety alone showed improvement in anxiety with positive yoga, and 40% of the studies assessing depression only showed positive intervention effects for depression.

## 2 Method

### 2.1 Search strategy

Literature search with “depression” OR “depressive disorder” OR “neurosis, depressive” OR “depressive symptoms” OR “major depressive disorder” AND positive yoga (“yoga” OR “yoga therapy” OR “yoga exercise” OR “yoga”) OR “yoga therapy” OR “yoga exercise” OR “yoga practice” OR “yoga intervention” OR “mindfulness yoga” in PubMed, Web of Science, CNKI database, and other databases to complete the search. The search period was from database creation to November 2022. The original results from the search were literature-weighted and screened, and relevant studies were supplemented with references from the included studies when necessary.

### 2.2 Selection criteria

Case-randomized controlled study and own-control study. Patients with depression diagnosed by HAMD, SDS or EPDS or other diagnoses were included. The experimental group was given positive yoga treatment (breathing, movement, meditation), and the control group was given conventional intervention. The severity of depression was assessed by HAMD (Hamilton Depression Scale), SDS (Self-Rating Depression Scale) or EPDS (Edinburgh Postnatal Depression Scale) or other types of depression measurement scales.

### 2.3 Data extraction

Regarding various types of data, such as patient (age, gender, diagnosis) method (randomized, blinded) intervention (type, duration, frequency of yoga) control group intervention (type, frequency, duration) outcome indicators, etc., will be done by two evaluators, and in case of dispute a third evaluator will be involved in the discussion to reach consensus.

### 2.4 Quality assessment

The risk of bias assessment tool of Review Manager 5.4 was used to evaluate the included studies in the following six aspects: ① generation of random assignment scheme; ② concealment of assignment scheme; ③ implementation of blinding method; ④ completeness of outcome data; ⑤ no selective reporting of results; ⑥ other sources of bias. “Low risk” indicates low risk of bias, “high risk” indicates high risk of bias, and “Unclear risk” indicates that the literature does not provide enough or uncertain information for bias assessment. To ensure the objectivity and scientific validity of the meta-analysis, the quality scores of the literature were conducted independently by two investigators, and the scores were compared with each other after completion.

### 2.5 Data analysis

Meta-analysis and heterogeneity tests were performed using Review Manage 5.4 software for the final screened literature using the random effects model (Random effects, *RE*), and the combined effect sizes of continuous variables were expressed as standardized mean difference (*SMD*). For continuous data, weighted mean difference (*MD*) was used for analysis if the differences in mean values of outcome indicators were small or if the same measurement tools were used; if different measurement tools were used for the same variables or if the differences in mean values of outcome indicators were large, standardized mean difference (*SMD*) was used for analysis, and 95% confidence intervals were calculated for all analyses. When heterogeneity existed among the literature for meta-analysis, the sources of heterogeneity were analyzed using subgroup analysis, and finally, funnel plots and forest plots were used for publication bias detection, with *p* < 0.05 being considered a statistically significant difference. Heterogeneity was tested by *Q* test and *I*^2^ test, and publication bias analysis was performed by Egger test. If *p* > 0.01 and *I*^2^ < 50%, a small heterogeneity was considered, a fixed-effects model (*FE*) was used; if *p* < 0.10 and *I*^2^ > 50%, a large heterogeneity was considered, a random-effects model (*RE*) was used, and further sensitivity analysis and Meta regression analysis were used. Subgroup analysis was used to compare the differences in performance of the positive yoga exercise program on different depressed subjects.

**Figure 1:**
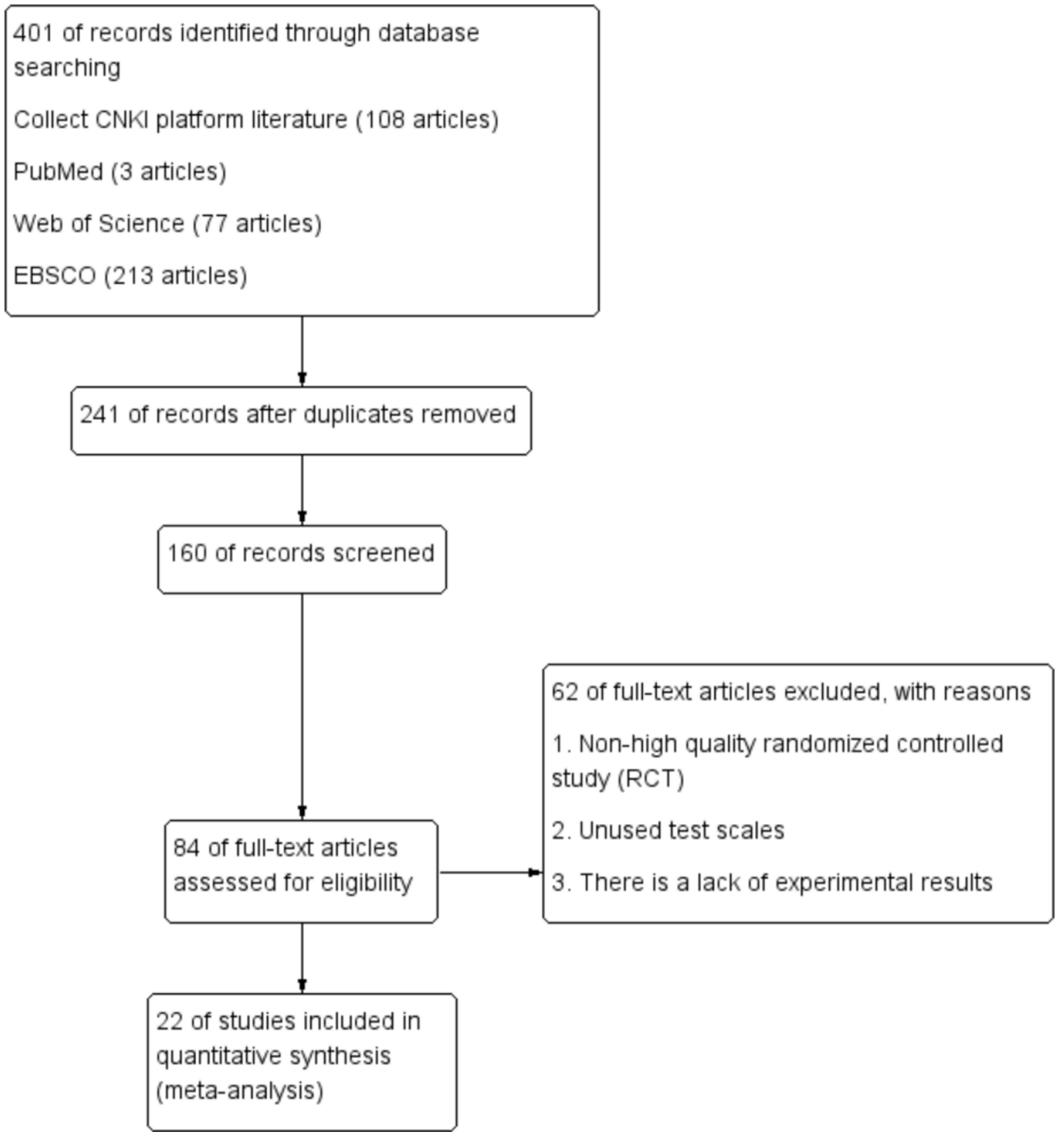
Literature screening flow chart.

A total of 401 relevant papers were obtained by database search, including 293 in English and 108 in Chinese (Figure 1). After reading the titles and/or abstracts to exclude those that obviously did not meet the inclusion criteria, 241 papers that were repeatedly published, crossed over, and did not meet the inclusion criteria were excluded, 160 papers were screened, and 84 papers were assessed to be eligible, and then after finding reading the full text and quality evaluation, 62 of them were excluded due to: ① non-high quality randomized controlled studies (RCT) ② not using test scales ③ missing experimental results. Therefore, they were removed, and 22 qualified papers were finally included.

## 3 Results

### 3.1 Characteristics and quality of the included studies

A total of 2,216 patients, including 1,101 in the yoga intervention group and 1,115 in the control group. Nine studies were interventions for postpartum depression, 10 studies were mindfulness yoga interventions for patients with common depression, and 3 studies were interventions for patients with other diseases and symptoms of depression. Six studies used the Edinburgh postnatal depression scale (EPDS), nine studies used the Self Rating Depression Scale (SDS), and five studies used the Hamilton Depression Scale (HAMD). Other outcome measures included the Hospital Anxiety and Depression Scale (HADS). The general situation and baseline characteristics of the included studies are detailed in Table 1.

**Table 1:** Basic characteristics of included literature.

References	Age	*N*		Intervention programs		Outcome indicators
		EG	CG	EG	CG	
Yang (2007)	19 ~ 23	30	30	MY		SDS
Lin et al. (2010)	18 ~ 45	30	30	MY	CC	SDS
Wang et al. (2014)	EG:23 ~ 29 CG:21 ~ 32	20	20	MY	CC	SDS
Bao et al. (2015)	19 ~ 24	24	24	MY	CC	SDS
Qiu et al. (2017)	19 ~ 43	95	105	MY	CC	EPDS
Niu and Guo (2017)	19 ~ 43	40	40	MY	CC	HAMD
Shu et al. (2019)	20 ~ 45	43	43	MY	CC	EPDS
Li and Lin (2019)	18 ~ 35	50	50	MY	CC	EPDS
Li et al. (2020)	20 ~ 40	41	41	MY	CC	EPDS
Gui (2020)	EG:21 ~ 39 CG:22 ~ 38	47	47	MY	CC	SDS
Gao et al. (2020)	22 ~ 40	42	42	MY	Venlafaxine	HAMD
You (2020)	EG:30 ~ 68 CG:31 ~ 70	189	189	MY	CC	HADS
Chen and Ye (2020)	EG:28 ~ 53 CG:25 ~ 52	26	30	MY	CC	HAMD
Li et al. (2021)	18 ~ 65	32	32	MY	CC	HAMD
Zhao and Xu (2021)	EG:28 ~ 55 CG:26 ~ 55	59	59	MY	CC	HAMD
Liang et al. (2021)	13 ~ 18	35	35	MY	CC	SDS
Liu and Zhang (2022)	EG:22 ~ 52 CG:22 ~ 51	45	45	MY	CC	SDS
Zhang et al. (2021)	29 ~ 57	38	38	MY	CC	HADS
Yang et al. (2021)	EG:21 ~ 34 CG:22 ~ 35	60	60	MY	Yoga Nidra	EPDS
Liu and Zhang (2022)	22 ~ 40	43	43	MY	CC	EPDS
Sun and Zheng (2022)	19 ~ 45	49	49	MY	CC	SDS
Wang et al. (2022)	EG:29 ~ 45 CG:31 ~ 46	63	63	MY	CC	SDS

MY, Mindfulness Yoga; CC, Conventional Care.

### 3.2 Sensitivity analysis

**Figure 2:**
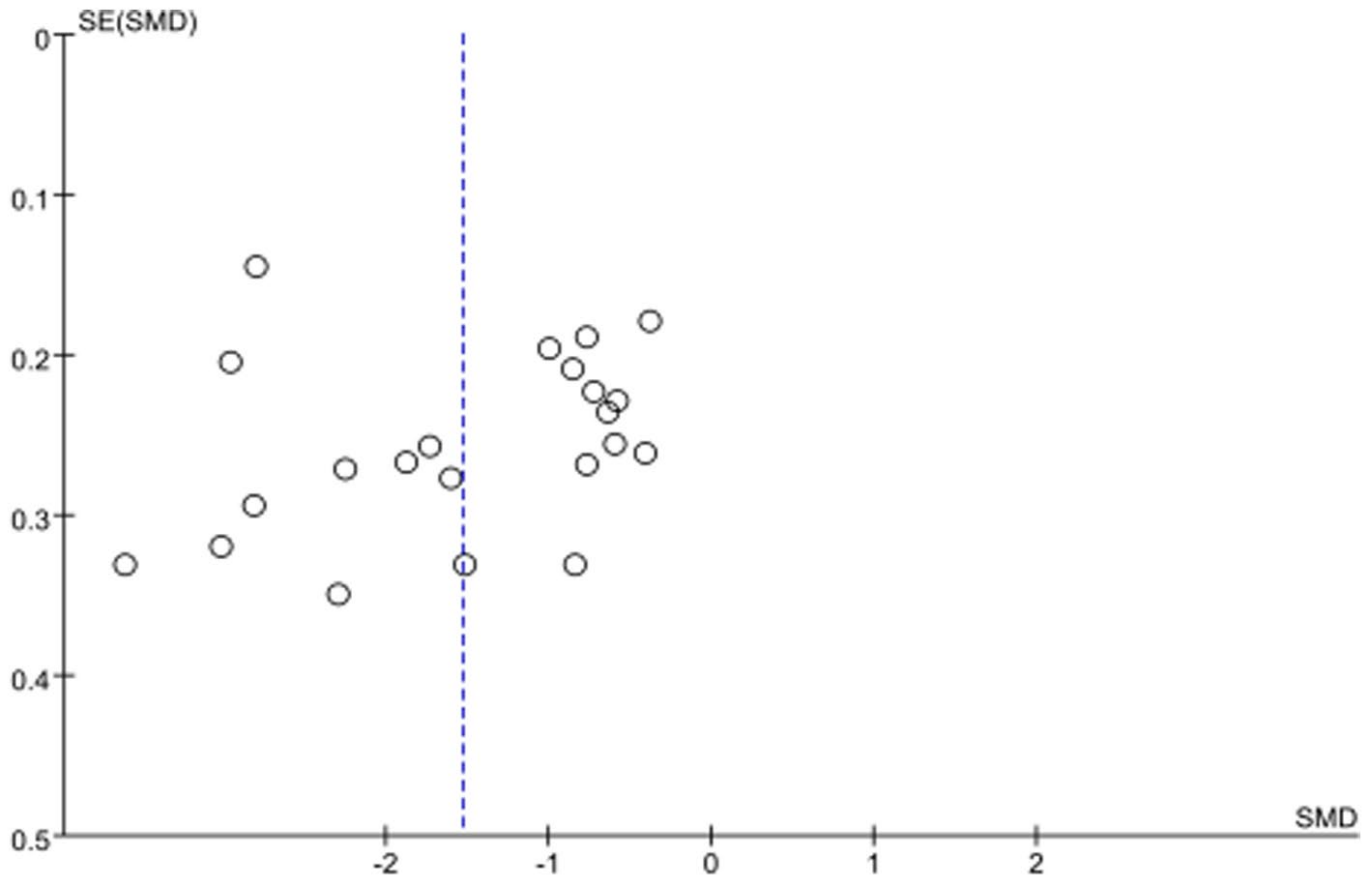
Publication bias funnel plot.

In this study, the sensitivity analysis was performed by excluding the literature one by one. After the meta-analysis was performed again, the results were compared with the previous data. It was found that the change of the combined effect results of each literature was not obvious, indicating that the results of the meta-analysis were credible. Only one paper intersected with the vertical line, indicating that the results of this study were stable. The results showed that the distribution of each study was uniform, suggesting that the possibility of publication bias was small, and the sample size was large, and the standard error was small, as shown in Figure 2.

### 3.3 Risk of bias evaluation of included studies

**Figure 3:**
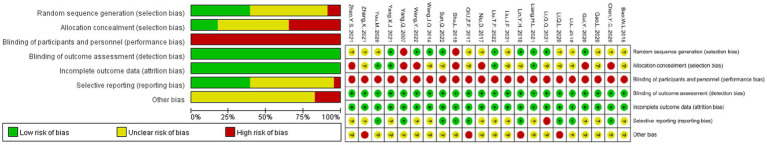
Bias risk assessment of included studies.

According to the bias risk assessment method recommended by the Cochrane assistance network. The baselines of the 22 studies included were comparable, but all had different levels of bias (Figure 3). Two of the 22 studies did not mention that randomization was rated as high risk (Yang, 2007; Shu et al., 2019). Nine studies reported that the sampling method was cluster sampling and allocation, and were therefore rated as low risk. Eight studies assigned exercise programs according to preferences or gender when grouping interventions, making it possible for subjects or researchers to predict allocation results, making it difficult to achieve allocation concealment, and thus being rated as high risk. In terms of blinding of subjects and staff, 22 studies were rated as high-risk because the intervention methods of the experimental group and the control group were completely different, and the control group had no intervention measures. Therefore, it could not comply with the double-blind principle, resulting in selection and implementation bias, and the outcome might be affected by the lack of blinding. Therefore, all studies assessed the risk of bias in incomplete outcome data as low risk. One study was rated as high risk because it difficult to obtain or replicate (Li et al., 2021). Finally, the three studies described the limitations at the end, and were therefore rated as high-risk (Lin et al., 2010; Li et al., 2020; Zhang et al., 2021). The remaining studies were unclear about other shifts due to insufficient information judgment.

### 3.4 Meta-analysis of the effect of yoga exercise on depression level

**Figure 4:**
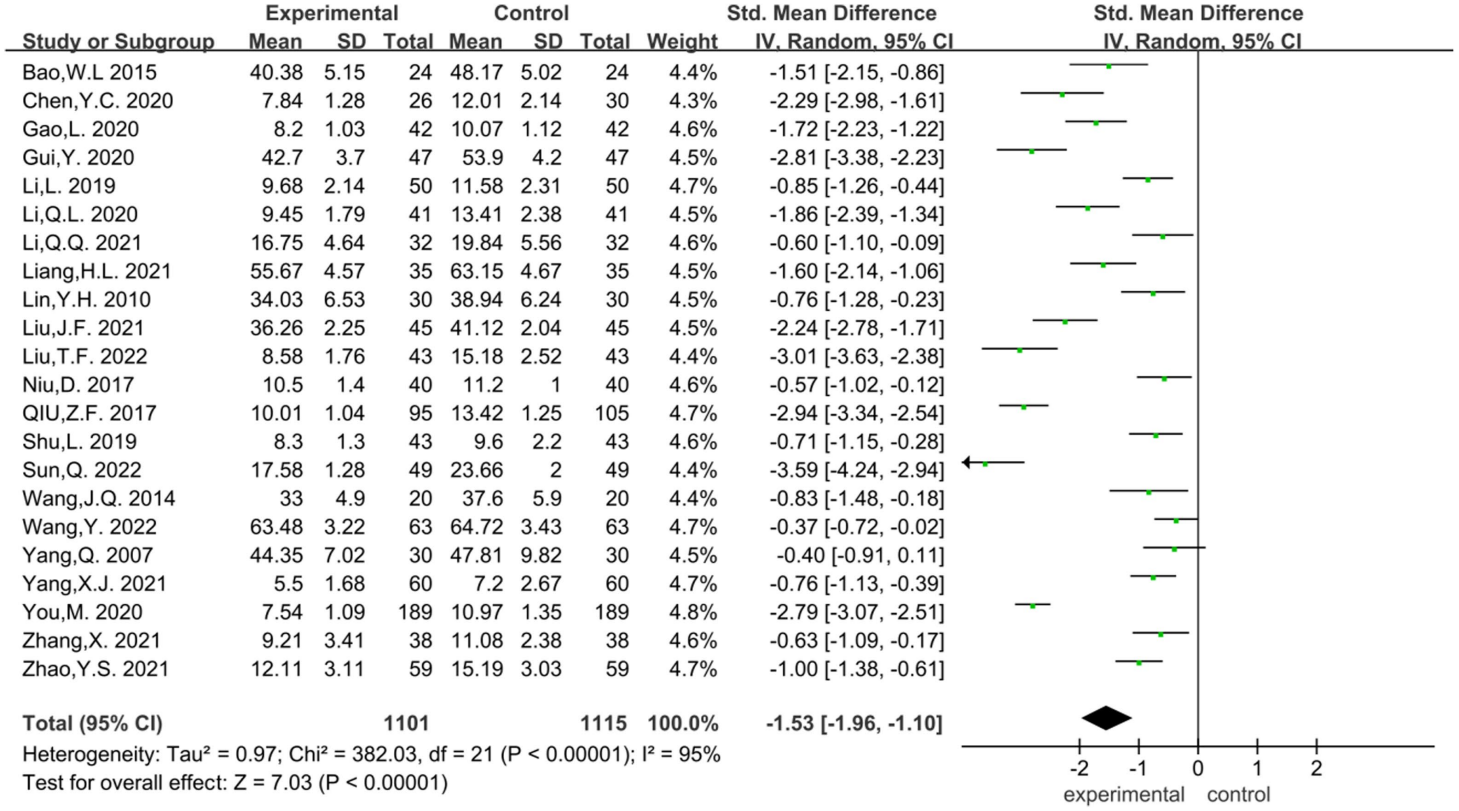
Meta-analysis forest plot of the effect of yoga exercise on depression level.

In the 22 literatures on the effect of mindfulness yoga intervention on depression level, the heterogeneity test showed that (Figure 4): heterogeneity coefficient (*p* < 0.00001, *I*^2^ = 94%), indicating that there was a strong heterogeneity between the control experiments included in the meta-analysis, and the measurement tools of each study were different. Therefore, *SMD* was selected as the combined statistic, and the random effect model (*RE*) was used to combine the effect quantity. The overall effect size *SMD* = −1.53, 95%*CI* was [−1.96, −1.10], and the combined effect size test *Z* = 7.03, *p* < 0.00001. According to Cohen’s research explanation, 0.8 or more is a large effect size, and *p* < 0.05, indicating that the combined effect size of multiple sets of data is statistically significant. The data showed that the level of depression in the mindfulness yoga intervention group was significantly lower than that in the control group, which could reduce the standard deviation by 1.53 times. That is, the use of mindfulness yoga could better improve the effect of depressive symptoms in patients, which was statistically significant. However, due to the substantial heterogeneity between the studies, sensitivity analysis was performed, and the included studies were excluded one by one. However, due to the substantial heterogeneity between the studies, sensitivity analysis was performed, and the included studies were excluded one by one. When the study of Bao et al. (2015) or Liang et al. (2021) was deleted, the heterogeneity was significantly increased (*I*^2^ = 95%, *p* < 0.00001). When you’s research report is deleted, the heterogeneity is significantly reduced (*I*^2^ = 93%, *p* < 0.00001).

### 3.5 Subgroup analysis of moderating variables

According to the heterogeneity of the overall effect size test, further subgroup analysis of the regulatory variables is needed to explore the source of heterogeneity. In this study, four adjustment variables, including outcome measurement index, weekly intervention frequency, intervention duration and experimental subject (prevalence of disease) were set up and tested in subgroups (Table 2).

**Table 2:** The results of the effect of moderating variables on depressive symptoms in mindfulness yoga programs.

Regulated variable	Heterogeneity test			Subgroup	Number of studies	*95% CI*	Two-tailed test	
	*χ^2^*	*P*	*I* ^2^				*Z*	*P*
Outcome indicators	59.87	<0.00001	95%	SDS	9	−5.80 [−7.72, −3.89]	5.94	<0.00001
				EPDS	6	−3.13 [−4.42, −1.85]	4.77	<0.00001
				HAMD	5	−2.47 [−3.54, −1.37]	4.35	<0.00001
				HADS	2	−2.79 [−4.29, −1.29]	3.64	0.0003
Freq. (times/week)	11.8	0.008	74.6%	1	1	−0.33 [−0.84, 0.18]	1.26	0.21
				2	5	−0.93 [−1.49, −0.37]	3.26	0.001
				3–4	10	−1.53 [−2.01, −1.05]	6.12	<0.00001
				5	6	−1.20 [−1.87, −0.53]	3.49	0.0005
Duration (weeks)	17.8	0.0005	83.1%	8	5	−1.01 [−1.62, −0.38]	3.21	0.001
				9–10	4	−0.26 [−0.64, 0.12]	1.35	0.18
				12	8	−1.69 [−2.31, −1.07]	5.36	<0.00001
				16	5	−1.33 [−2.09, −0.56]	3.38	0.0007
Experimental subject	1.61	0.45	0%	With other diseases	3	−1.89 [−3.23, −0.56]	2.78	0.005
				Ordinary depression	10	−1.21 [−1.71, −0.71]	4.73	<0.00001
				Postpartum depression	9	−1.68 [−2.34, −1.02]	4.96	<0.00001

#### 3.5.1 Outcome indicators

The 22 articles were divided into four groups: SDS, EPDS, HAMD and HADS according to different scales. *MD* was selected as the statistic. According to the results, the effect size of *RE* was combined. The effect size of the four groups had high heterogeneity (*I*^2^ = 95%), indicating that the measurement of different outcome indicators had a certain impact on the relationship between exercise intervention and depressive symptoms. Among them, SDS had the greatest effect on the results of depressive symptoms *d* = −5.80 (*p* < 0.00001), 95%*CI*: [−7.72, −3.89]; the second is the EPDS measurement index, the effect size *d* = −3.13 (*p* < 0.00001), 95%*CI*: [−4.42, −1.85]. Among them, the effect size of HADS was *d* = −2.79, 95% *CI*: [−4.29, −1.29] (*p* < 0.00001); the effect size of HAMD was the smallest, *d* = −2.47, 95% *CI*: [−3.54, −1.37] (*p* < 0.00001).

#### 3.5.2 Weekly intervention frequency

The study was divided into four groups according to the frequency of weekly exercise intervention, and *SMD* was selected as the statistic. According to the results, the combined effect of RE was used. The effect size of the four groups had moderate heterogeneity (*I*^2^ = 74.6%), indicating that different weekly intervention frequencies had a certain effect on depressive symptoms. Among them, the 3–4 times per week intervention produced the largest effect size of *d* = −1.53 (*p* < 0.00001), 95%*CI*: [−2.01, −1.05] for depressive symptom outcomes; followed by the 5 times per week exercise intervention with an effect size of *d* = −1.20 (*p* = 0.0005), 95%*CI*: [−1.87, −0.53]; in addition, the 2 times per week intervention had an effect size was *d* = −0.93 (*p* = 0.001), 95%*CI*: [−1.75, −0.12]; in addition, the effect size for the 2 times per week intervention was *d* = −0.93 (*p* = 0.001), 95%*CI*: [−1.75, −0.12]. The smallest effect size was for the once-weekly exercise intervention, *d* = −0.33, 95%*CI*: [−0.84, 0.18], which was not significant because *p* = 0.21 > 0.05.

#### 3.5.3 Exercise intervention duration

The 22 studies were divided into 4 groups based on the exercise intervention period, and *SMD* was selected as the statistic, and the effect size was combined using RE based on the results, with a high degree of heterogeneity (*I*^2^ = 83.1%) across the 4 groups, indicating that there was an effect of different weekly intervention periods on depressive symptoms. Among them, the 12-week intervention produced the largest effect size of *d* = −1.69 (*p* < 0.00001), 95%*CI*: [−2.31, −1.07] in improving depressive symptoms outcomes; followed by the 16-week intervention group with an effect size of *d* = −1.33 (*p* = 0.0007), 95%*CI*: [−2.09, −0.56]; in addition, the 8-week intervention group had an effect size of *d* = −1.01 (*p* = 0.001), 95%*CI*: [−1.62, −0.38]. Finally, the smallest effect size was in the 9-10-week intervention group with *d* = −0.26, 95%*CI*: [−0.64, 0.12], which was not significant because *p* = 0.18 > 0.05.

#### 3.5.4 Experimental subject

Due to the heterogeneity between the studies, the depressive symptoms were analyzed according to the subgroup of the experimental subjects. The results were as follows: on the index of the experimental subjects, the scores of postpartum depression patients in the intervention group were lower than those in the control group (*SMD* = −1.68, 95%*CI* = [−2.34, −1.02], *p* < 0.00001). The depression tendency of patients with other diseases in the intervention group score was lower than that in the control group (*SMD* = −1.89, 95%*CI* = [−3.23, −0.56], *p* = 0.005); according to the general depression patients, the intervention group scores were lower than the control group (*SMD* = −1.21, 95%*CI* = [−1.71, −0.71], *p* < 0.00001). However, the *p* value in Heterogeneity Test was not significant, indicating that experimental subject was not the source of heterogeneity.

## 4 Discussion

With the acceleration of the pace of life in today’s society, people’s pressure on learning, employment and other aspects has increased dramatically, which has caused a greater impact on mental health. Depression is a common mental illness in clinic, which is characterized by continuous low mood, high incidence and disability rate, causing serious economic burden to patients (Zhang, 2017). It may occur at all ages, posing a serious threat to the public’s mental health, and the cost of treatment is high (Wu, 2020). The disease has a long course of disease and is prone to recurrence. It is easy to produce other negative emotions. Negative emotions continue to accumulate, seriously affecting work and life, leading to decreased social function, resulting in a vicious circle (Guo et al., 2021). It is expected that 2030 will become the first cause of suicide (Friedrich, 2017).

A nationwide epidemiological survey (Huang et al., 2019) showed that the lifetime prevalence of depression in China was 3.4%. The main treatment of the disease is drug therapy, but only the application of drug therapy is limited (Kang and Sheng, 2020). European Psychiatric Association recommends the combination of drug therapy and psychotherapy (Probst et al., 2020). “Mindfulness” originated from the Eastern Buddhist culture and has developed from meditation and meditation to a systematic psychotherapy (Williams et al., 2020). This paper systematically analyzes the effect of mindfulness therapy on depression, anxiety symptoms and sleep quality in patients with depression. Mindfulness therapy is a common method for the treatment of mental disorders. It has formed a relatively mature system and has a significant effect in the treatment of mental disorders such as depression, anxiety and personality disorders (Spinhoven et al., 2017). As a non-drug therapy for mental health intervention, applying mindfulness therapy to patients with depression can help alleviate their anxiety and depressive symptoms, reduce the occurrence of rumination, reduce suicidal willingness, improve mindfulness skills, and promote disease recovery (Xia, 2020).

Of the 22 RCTs selected in this study, one was self-controlled and the rest were randomized controlled studies. Compared with the non-yoga treatment group, the score of depression was significantly lower in the yoga treatment group. Compared with simple meditation, meditation, and complete yoga (exercise, breathing, meditation) combined with mindfulness, the improvement of depression was more significant. Yoga posture exercise promotes blood circulation and effectively improves sleep. Respiratory regulation can stabilize autonomic nerves, relieve stress and eliminate mental tension. Mindfulness meditation practice can make patients with depression calm, reduce depression and anxiety, and concentrate. There are still some deficiencies in the literature included in this study. The level of depression in the experiment is not strictly controlled, and some depressed patients have physical diseases (Hua et al., 2018), which may seriously interfere with patients’ yoga practice, especially cancer and bone and joint diseases. There are also many differences in the types of depression. The depression assessment scale may not truly reflect the degree of depression in patients due to different cultures. In this study, a meta-analysis method was used to quantitatively evaluate the improvement of yoga exercise on mental health.

### 4.1 The intervention mechanism of mindfulness yoga on mental health

According to scientific experiments, when people enter the state of meditation, the activity of the brain will show regular brain waves. At this time, the role of the neocortex of the brain that governs intellectual and rational thinking will be inhibited, while the autonomic nerves that dominate animal instincts and self-will and cannot be controlled, as well as the role of the brainstem and the lower part of the thalamus that are responsible for adjusting hormones, will become active (Qi et al., 2020). When entering the yoga meditation state, the practitioner must slow down the muscles, cells and blood circulation of the whole body, and finally make the meditator obtain inner peace and tranquility.

Yoga uses complete breathing, that is, the combination of abdominal breathing and chest breathing. This breathing method is analyzed from a physiological point of view. More oxygen is taken in when inhaling, and more carbon dioxide and toxic substances are discharged when exhaling, which can make each cell of the body work better and more fully. With each breath, the diaphragm will also fluctuate regularly, and it can also play the role of massage and reconciling internal organs (Smith et al., 2023). From a psychological point of view, because attention is focused on breathing, it can make the practitioner calm and mentally stable. At the same time, more oxygen is delivered to the brain, which also increases the attention of the practitioner and enhances the brain’s ability to coordinate bodily functions (Xiong and Li, 2014).

### 4.2 The effect of mindfulness yoga on depressive symptoms in patients with depression

The results of meta-analysis showed that mindfulness therapy was helpful to improve depressive symptoms in patients with depression. However, there is a large heterogeneity in depression indicators. Subgroup analysis was performed on the included studies according to the research objects and evaluation scales. Among the subjects, the depressive symptoms were relieved after the intervention of mindfulness therapy, but the heterogeneity was large, which may be related to the inclusion of patients with depression in different diagnostic grades. Because only individual studies described detailed diagnostic grades, it was impossible to further analyze the application of mindfulness therapy to patients with depression in different diagnostic grades. In terms of evaluation scales, after combining the effect values of SDS, EPDS and HAMD, it was found that the heterogeneity was reduced, and the EPDS score was significantly reduced, suggesting that the inconsistency of the evaluation scales used among the studies may be the source of heterogeneity. The unity of the evaluation methods of outcome indicators can be the focus of future research. Due to the different evaluation time used in each study and the small number of literatures included, the evaluation time was not combined and analyzed. All studies compared the efficacy within 3 weeks after the intervention, and the results showed that mindfulness therapy helped to alleviate depressive symptoms in patients with depression (*p* < 0.00001). Some studies were followed up for more than 6 weeks, and the results showed that the long-term effect of mindfulness therapy on relieving depressive symptoms was not obvious (*p* = 0.52). To this end, Segal et al. (2020) proposed that future research should pay more attention to the duration of intervention time, and evaluate the medium and long-term intervention effects of mindfulness therapy by prolonging the intervention time.

### 4.3 The intervention effect of mindfulness yoga on improving mental health at home and abroad

Foreign studies have shown that in the field of physiology and psychology, researchers have been able to perform various physiological tests such as electromyography, electroencephalogram and respiratory status before and after yoga training, and demonstrate that yoga training can reduce cortisol content in the body (Amitani et al., 2022), relieve psychological pressure (Schmalzl and Kerr, 2016), and improve people’s mental health (Wang et al., 2020). Foreign scholar (Daley et al., 2015) believes that yoga practice can make individuals feel happy and satisfied (Kuyken et al., 2015), which is conducive to physical health. Prenatal yoga exercise has the most significant effect on preventing maternal stress and anxiety (Niu and Guo, 2017; Gui, 2020; Miao et al., 2023b), and has a positive effect on emotional improvement (Zhou et al., 2021). Domestic scholars believe that each action to complete yoga needs to be based on mindfulness meditation and listening to achieve the realm of physical and mental integration (Shu et al., 2018; Wu and Feng, 2023). After practicing yoga, practitioners can feel physical and mental pleasure. Long-term practice can cultivate a natural state of mind (Chen and Ye, 2020), which is of great help to alleviate the release of negative emotions such as psychological anxiety and depression. Liu and Zhang (2022) selected 86 cases of second-child pregnant women as the research object, and randomly divided them into two groups. The patients in the control group were given routine psychological nursing, and the patients in the study group were given mindfulness yoga training based on the control group. The study found that mindfulness yoga can effectively alleviate the generation and accumulation of depression during pregnancy in second-child pregnant women, shorten the labor process of pregnant women, and have a significant effect on PPD prevention and depression reduction. Du and Zhang (2005) and Shan et al. (2017), etc. have selected people with depression as experimental subjects for yoga practice. The results show that there are very significant differences in anxiety and depression factors before and after practice. It is concluded that yoga practice can alleviate depression.

## 5 Conclusion

Limitations: (1) Only original studies in Chinese and English were included in this meta-analysis, which increased the risk of incomplete literature inclusion. (2) The evaluation time of the included literature was different, and there was a problem of short evaluation time. Because only 22 original studies were included, some factors affecting the severity of depression, such as the diagnosis and classification of depression and the use of depression drugs, could not be further explored. (3) The specific measures of mindfulness intervention adopted in each study are not the same, and the interpretation of the results needs to be cautious.

In summary, mindfulness meditation training combined with group yoga intervention for patients with depression can effectively improve the depression and anxiety status of patients, improve the cognitive level of patients with depression, and improve the quality of life, which is worthy of clinical application. Colleges, enterprises and obstetrics and gynecology hospitals should popularize mindfulness yoga and configure corresponding instructors or teachers to help improve the physical quality of the sick group and reduce the level of depression and anxiety. To a certain extent, it can also alleviate and prevent depression.

## Data availability statement

The original contributions presented in the study are included in the article/supplementary material, further inquiries can be directed to the corresponding authors.

## Author contributions

YY: Conceptualization, Data curation, Formal analysis, Investigation, Software, Visualization, Writing – original draft. DC: Funding acquisition, Methodology, Project administration, Resources, Supervision, Writing – review & editing. TL: Methodology, Project administration, Supervision, Writing – review & editing. WG: Investigation, Supervision, Writing – review & editing.
